# Immobilization of Porcine Trypsin in Superparamagnetic Nanoparticles: Enzyme Activity and Stability

**DOI:** 10.1021/acsomega.5c00797

**Published:** 2025-06-02

**Authors:** Isabella F. S. Aversa, Marcello H. S. Cavalcanti, Thalles M. Pereira, Alexandre A. de Castro, Olga L. Tavano, Yara L. Coelho, Luis H. M. da Silva, Luiz F. Gorup, Teodorico C. Ramalho, Luciano S. Virtuoso

**Affiliations:** † Colloid Chemistry Group, Chemistry Institute, 74347 Federal University of Alfenas (UNIFAL-MG), 700 Gabriel Monteiro da Silva street, Alfenas, MG 37130-000 , Brazil; ‡ Department of Chemistry, 67739 Federal University of Lavras, Lavras, MG 37200-000, Brazil; § Advanced Thermokinetics of Molecular Systems (ATOMS) Group, Department of Chemistry, 28120 Federal University of Viçosa, PH Rolfs, Viçosa, MG 36570900, Brazil; ∥ Department of Chemistry, Federal University of Juiz de Fora (UFJF), Juiz de Fora, MG 36036-900, Brazil

## Abstract

This study explores the immobilization of porcine trypsin (PT) on superparamagnetic iron oxide nanoparticles (SPIONs) via adsorption, with a focus on both immobilization conditions and a comprehensive energetic evaluation—an aspect often neglected in the literature. Isothermal titration calorimetry (ITC) revealed that interactions at pH 8.0 are energetically favorable, with a Δ*H*° of −43.0 kJ mol^–1^, suggesting robust noncovalent interactions. PM6 calculations confirmed the stability of the adsorption. The resulting nanobiocatalyst showed over 75% activity recovery at pH 8.0 and retained around 40% activity after four reuse cycles, demonstrating its efficiency and robustness. The detailed energetic analysis provides critical insights for optimizing immobilization processes, impacting cost and technical feasibility, and advancing the understanding of enzyme-support interactions for scalable applications in biocatalysis.

## Graphical abstract

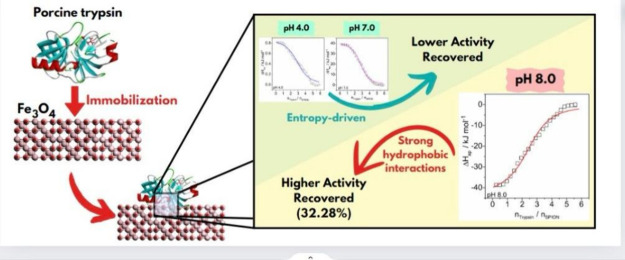

## 1 Introduction

Enzymes are a catalyst with high potential for application in biotechnological processes because they are highly selective biocatalysts.^1,2^ In recent decades, the proteolytic enzyme has been widely used as a biocatalyst in industrial processes.^3^

Trypsin (EC 3.4.21.4) is a serine endopeptidase, also known as serine protease, and its high proteolytic potential has been applied in recent decades in the food and beverage industries to improve and facilitate the process, increasing the tenderness of meats and pasta quality, in the cold stabilization of beer, in addition to the production of protein hydrolysates.^4–6^ Although trypsin has a high biotechnological potential, they are not chemically stable and quickly loses its biological activity.^7^ Due to the impossibility of their reuse in soluble (free) form and the high costs, the application of soluble enzymes on a large scale is very limited.^8^ A solution to overcome these drawbacks has been the immobilization of these enzymes on solid supports.^9,10^

Enzyme immobilization on support materials has been a very common strategy for most applications, being a method that keeps protein molecules confined or located in a certain defined region of space, with the protection of their catalytic activities.^11,12^ Enzymes could be immobilized by covalent bond or physical entrapment on a variety of solid supports, such as polymeric matrices,^13^ porous materials, carbon nanotubes,^14^ membranes,^15^ and magnetic materials.^16^

Among the many different types of support that have been employed in the immobilization of enzymes, the use of nanomaterials, especially those with magnetic properties, has recently gained prominence.^17^ In particular, the application of superparamagnetic nanoparticles of magnetite (Fe_3_O_4_) as a support for enzyme immobilization has been of great interest, due to their low toxicity, large available surface area, low cost, and their superparamagnetic properties.^18^ Magnetic nanoparticles below a certain critical size can exhibit superparamagnetic behavior.^19^ The critical Fe_3_O_4_ particle size, which results in a transition from a multiple-domain to a single-domain structure, necessary to attain superparamagnetism, is dependent on several factors but has been broadly estimated to be below 20 nm.^20^ Superparamagnetism gives iron oxide nanoparticles (SPIONs) great simplicity of isolation from complex multiphase media by the application of an external magnetic field, and easy dispersion after removal of the external field.^21^ Due to this resource, solid is separated under the influence of an external magnetic field, facilitating the separation and recovery of the enzyme, eliminating undesirable steps, such as the need for centrifugation, sample dilution, and loss of support during washes. As the magnetic separation is fast and efficient, there is also a time saving.^22^

Recent studies have primarily focused on structural analyses postimmobilization, but there is a need for further research into one-step physical adsorption from a thermodynamic perspective.^23–25^ In this work, a nanobiocatalyst formed by the adsorption of porcine trypsin with SPIONs was prepared and broadly characterized, and its catalytic activity was evaluated. The SPION-trypsin interaction was thermodynamically characterized with the aim of contributing to an understanding of events at the nanoparticle-enzyme interface. The pH control conditions and surface charge properties of both trypsin and SPIONs in the immobilization process were investigated. It was shown that the immobilized enzyme can be separated from the reaction medium easily, quickly, and efficiently by an external magnetic field. The enzymatic properties of the immobilized enzyme were then investigated in terms of the proteolytic activity recovered after the immobilization process, the amount of enzyme effectively immobilized on the support surface, the enzyme desorption rate, and the operational stability rate of several reuse cycles.

## 2 Experimental Section

### 2.1 Materials

Iron (II) sulfate heptahydrate (99%, Sigma Aldrich, USA), anhydrous iron (III) chloride (99%, Vetec, Brazil), and sodium hydroxide (99%, Sigma Aldrich, USA). In the synthesis of SPIONs, nitrogen gas (N_2_) (99%, White Martins, Brazil) was used. For immobilization and evaluation of enzymatic activity, trypsin from porcine pancreas - EC 3.4.21.4 (Sigma Aldrich, USA, T-0303, Type IX-S, lyophilized, 13,000–20,000 BAEE units/mg protein). The total protein content of the trypsin material was tested using the Kjeldahl method,^26^ with protein calculated as nitrogen × 6.25, confirming 100% protein content. N_α_-Benzoyl-l-arginine 4-nitroanilide hydrochloride (L-BApNA), (Sigma Aldrich, USA), Dimethylsulfoxide (DMSO), (Sigma Aldrich, USA), acetic acid (99%, Sigma Aldrich), Tris-HCl buffer (Sigma Aldrich, USA), Hydrochloric acid (37%, Sigma Aldrich, USA), Sodium phosphate buffer (Sigma Aldrich, USA) and Sodium acetate (99%, Sigma Aldrich, USA). Deionized water obtained from a MILLI-Q water purification system (18.2 MΩ cm resistivity, Millipore, Bedford, USA) was used as a solvent in all steps.

### 2.2 Preparation of SPIONs

The synthesis of SPIONs was performed according to the coprecipitation method.^27^ For this, a solution of FeSO_4_·7H_2_O (s) was prepared by dissolving 3.5 g (0.0126 mol) of FeSO_4_·7H_2_O in 50 mL of deionized water. A second solution was prepared by dissolving 4.0 g (0.0246 mol) of anhydrous FeCl_3_ in 50 mL of deionized water. The two solutions were then mixed in a three-way round-bottomed flask (250 mL) and kept under moderate stirring under a nitrogen atmosphere. Then, a solution of 20 mL of 10 mol L^–1^ NaOH was dripped into the synthesis flask at a flow rate of approximately 5 drops per minute (0.2 mL min^–1^). The entire process, from the beginning of the sodium hydroxide dripping, lasted approximately 3 h. The SPIONs obtained were separated from the liquid reaction medium by the action of an applied external magnetic field and were subjected to successive washings with deionized water until the pH of the washing water became neutral. The product was then kept in an oven at 60 °C under vacuum for 24 h for drying, and later, it was characterized and used in the immobilization process.

### 2.3 Immobilization of Porcine Trypsin

Enzymatic immobilization was performed under different pH control conditions: pH 4.0, using 0.005 mol L^–1^ sodium acetate buffer; pH 7.0 using 0.100 mol L^–1^ sodium phosphate buffer; and pH 8.0, using 0.010 mol L^–1^ Tris-HCl buffer. These conditions were previously selected based on the zeta potential data of both SPIONs and trypsin at each pH. In general, immobilization at each fixed pH occurred from the addition of 0.0025 g of porcine trypsin to 10 mL of buffer solution. Next, 1.0 g of SPIONs was added to the mixture, which was stirred at room temperature for 24 h. After this period, the final product obtained, SPION@PT, was separated from the supernatant with the aid of a magnet, being subjected to at least 3 washes with deionized water. The material was then placed on filter paper and kept in a desiccator for drying, and then stored at a temperature of about 8 °C until used in subsequent steps.^28^ The amount of trypsin immobilized on the surface of SPIONs was determined by the difference between the amount of total trypsin used initially and the trypsin content determined in the supernatant at the end of the immobilization process. The enzyme content was determined by fluorescence using the Cary Eclipse Spectro (Varian) spectrofluorimeter. In general, the supernatant samples were prepared with 2 mL of deionized water and 50 μL of the supernatant. Then, they were excited at 279 nm, and the fluorescence was analyzed at the wavelength between 300 and 450 nm, with maximum intensity at 340 nm. From a previously constructed calibration curve, the trypsin concentration can be determined. All experiments were carried out in triplicate.

### 2.4 Hydrolytic Activity of Immobilized Trypsin

The enzymatic activity of free and immobilized trypsin was analyzed using the substrate benzoyl-DL-arginine-*p*-nitroanilide (BApNA),^29^ which generates *p*-nitroaniline as a product, which was monitored by a Thermo Scientific Evolution 60S UV–visible spectrophotometer at 410 nm. First, a BApNA solution (0.04 g BApNA, 1 mL of DMSO) was prepared. Then, 100 mL of 0.01 mol L^–1^ Tris-HCl buffer solution, pH 8.0, was slowly added, and the mixture was kept under a temperature control of 37.0 °C. Finally, 2 mL of a substrate solution was placed in a plastic cuvette, together with 0.2 mL of the enzyme solution (0.5 mg mL^–1^ in 0.01 mol L^–1^ Tris-HCl buffer, pH 8.0), in which the reaction mixture was incubated for 10 min in a thermostatic bath (Quimis, Q214-SC) at 37.0 °C. After the incubation time, 0.5 mL of 30% acetic acid solution was added, and the reading was performed on the spectrophotometer. A unit of activity is arbitrarily defined as an increase of 0.01 Abs/min.

### 2.5 Desorption Studies

The desorption study was performed by resuspending 20 mg of immobilized enzyme at pH 4.0, 7.0, and 8.0 in 2 mL of deionized water. This suspension was stirred for 5 min in an ultrasonic washer, and then the nanocatalyst was magnetically separated from the solution. This procedure was repeated 5 times for each pH value. The trypsin content present in the supernatant was determined by fluorescence spectroscopy according to the procedure described at the end of Section 2.3.

### 2.6 Enzyme-Nanoparticle Interaction

#### 2.6.1 Isothermal Titration Calorimetry (ITC) Experiments

The thermodynamics of SPION-PT interactions at 298.15 ± 0.00001 K were directly determined using a microcalorimeter (CSC-4200, Science Corp.) equipped with two removable calorimetric cells (reference and sample). The experimental setup involved the sequential addition of 24 injections (10 μL each) of a 1.79 × 10^–4^ mol/L PT solution, delivered with a Gastight Hamilton syringe (250 μL), into the sample cell initially containing 1.8 mL of SPION dispersion (0.1 mg/mL). The porcine trypsin (PT) solution and SPION dispersion were both prepared in sodium acetate buffer (0.005 mol/L, pH 4.0), sodium phosphate buffer (0.100 mol/L, pH 7.0), or Tris-HCl buffer (0.010 mol/L, pH 8.0). The reference cell was filled with 1.8 mL of deionized water.

During the experiment, the solution in the sample cell was continuously stirred at 180 rpm (3.0 s^–1^) using a gold helix stirrer to ensure thorough mixing. A 600 s interval between injections was maintained to allow the signal to return to baseline, ensuring accurate measurements. For control experiments involving dilution, the same procedure was applied, but the sample cell was filled with buffer in the absence of SPION. The raw data were recorded as a plot of power versus time.

The integration of the power versus time raw data provides the values of heat involved in each injection for both interaction (*q*_int_) and dilution (*q*_dil_) experiments. By subtracting *q*_dil_ from *q*_int_, the heat associated solely with the binding of PT to SPION was obtained for each injection (*q*_i_). Assuming the SPION-PT interaction follows the single set of identical sites (SSIS) model,^30^ the total heat accumulated in the binding event after *N* injections (*Q*_T_) is given by eq 1.

$$Q_{T}=\frac{\Delta H^{{}^{\circ}}V_{c}}{2K_{b}}[1+K_{b}[\mathrm{PT}{]}_{T}+nK_{b}[\mathrm{SPION}{]}_{T}-\sqrt{(1+K_{b}[\mathrm{PT}{]}_{T}+nK_{b}[\mathrm{SPION}{]}_{T}{)}^{2}-4n{K_{b}}^{2}[\mathrm{SPION}{]}_{T}[\mathrm{PT}{]}_{T}}]$$ 1

where Δ*H*° is the standard enthalpy change for the binding of PT to SPION, *V*_c_ is the effective cell volume, *K*_b_ is the binding constant, *n* is the stoichiometry of the complex formed, and [PT]_T_ and [SPION]_T_ are the total concentrations of PT and SPION in the sample cell after *N* injections. From the SSIS model, *q*_i_ can be expressed in terms of *Q*_T_ as shown in eq 2.

$$q_{i}=Q_{T}(i)-Q_{T}(i-1)+\frac{V_{\mathrm{inj}}}{V_{c}}\left(\frac{Q_{T}(i)+Q_{T}(i-1)}{2}\right)$$ 2

where *Q*_T_(*i*) and *Q*_T_(*i* – 1) are the total heat accumulated after the *i*th and (*i* – 1)­th injections given by eq 1, and *V*_inj_ is the injection volume. By fitting the experimentally obtained *q*_i_ values to eq 2, we determined the values of Δ*H*°, *K*_b_, and *n* were determined. Using *K*_b_ and Δ*H*°, the standard changes in Gibbs free energy (Δ*G*°) and entropy (Δ*S*°) were calculated by using eqs 3 and 4.

$$\Delta G^{{}^{\circ}}=-RT\ln K_{b}$$ 3

$$\Delta G^{{}^{\circ}}=\Delta H^{{}^{\circ}}-T\Delta S^{{}^{\circ}}$$ 4

#### 2.6.2 Modeling

The crystallographic structure of the porcine trypsin was obtained from the protein data bank (PDB), under the ID code: 2A31 and resolution: 1.25 Å.^31^ In the enzyme preparation protocol, the water molecules were removed, and the porcine trypsin was protonated at different pHs (4, 7, and 8) through the PDB2PQR web service (https://server.poissonboltzmann.org/pdb2pqr). The partial electrostatic charges were also calculated for all of the atoms of porcine trypsin at different pHs.

#### 2.6.3 Theoretical Calculations

Semiempirical methods are based on the Hartree–Fock formalism but perform several approximations by obtaining some parameters from empirical data. This category of techniques makes it possible the approach large systems with many atoms. Currently, it is commonly employed, allowing for the theoretical studies of proteins, DNA, enzymes, and other molecular systems with thousands of atoms. The semiempirical methods emerged in 1931 with the studies of Michael Polanyi and Henry Eyring,^32^ which address the quantum theory coupled to empirical results, producing satisfactory results. The PM6 semi-empirical method showed itself to be appropriate to perform the calculations of the adsorption of the porcine trypsin over magnetite (Fe_3_O_4_).

Summarizing, properties such as enthalpy of formation, dipole moment, ionization potential, angles, dihedral angles, and bond lengths are included. The reference database should be extended to a representative group of molecules that present good accuracy in the empirical results of their properties.^33^ It is important to notice that recent extensions of PM3 (PM6) seem to represent substantial improvements.^34^ Thus, the potential energy profile for the adsorption of porcine trypsin over Fe_3_O_4_ was computed through PM6 calculations, together with the elucidation of the electrostatic surfaces over the enzyme along different pHs. The immobilization of the enzyme over the material surface is illustrated in Figure 1.

**Figure 1:**
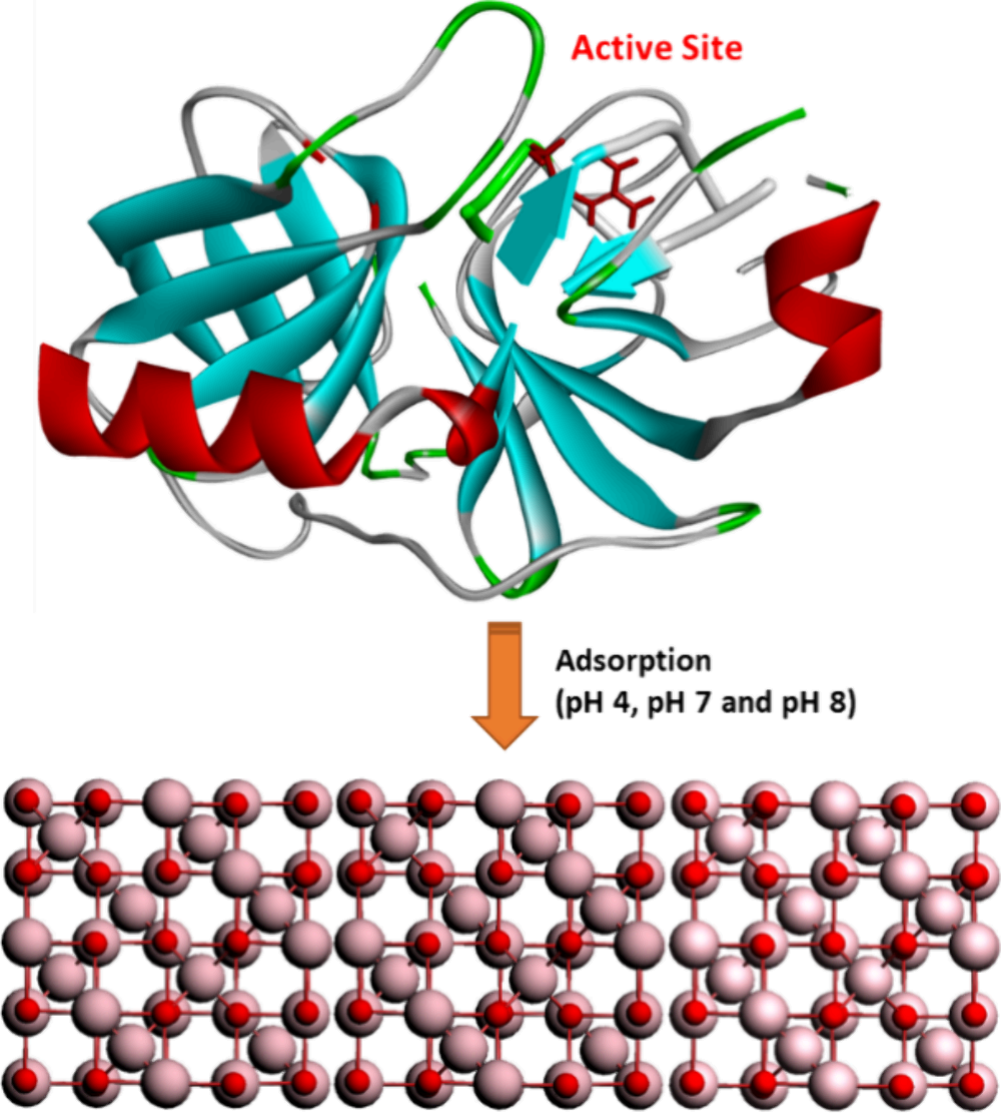
Immobilization of porcine trypsin on Fe_3_O_4_ (magnetite).

### 2.7 Catalytic Efficiency of the Immobilized Enzyme

First, a solution of BApNA was prepared by dissolving 0.04 g of BApNA in 1 mL of DMSO, followed by the addition of 100 mL of 0.01 M Tris-HCl buffer at pH 8.0. This substrate solution was maintained in a water bath at 37.0 °C until use. Next, 10 mL of the substrate solution was placed in a Duran bottle, along with 0.05 g of the immobilized material (SPION@PT). The reaction mixture was incubated in a thermostatic bath at 37.0 °C with agitation at 240 rpm for 10 min. After the incubation period, the biocatalyst was separated from the supernatant, washed three times with deionized water, and added to a fresh 10 mL aliquot of the substrate to initiate a new catalytic cycle. Subsequently, the supernatant from each reuse cycle was analyzed by using a spectrophotometer at 410 nm to determine the catalytic efficiency of the material.

### 2.8 Effect of Ionic Strength on the Immobilization and Activity of Trypsin

The study of the effect of ionic strength on the immobilization and activity of trypsin on SPIONs was conducted by adding 0.01 g of porcine trypsin to 20 mL of acetate buffer solution at pH 4.0 under different ionic strength conditions (2.5, 25.0, 50.0, and 100.0 mM). The methodologies described in Sections 2.3–2.5 of this work were followed.

### 2.9 Characterization

#### 2.9.1 X-ray Diffraction (XRD)

The nanoparticles of Fe_3_O_4_ prepared by the coprecipitation method used in the present work as an immobilization support were characterized using a RIGAKU diffractometer (model ULTIMA IV). For such, CuKα radiation (sealed tube) was used with parallel-beam instrumental optics with a wavelength (λ) of 0.15406 nm, a voltage of 40 kV, and a current of 30 mA. The measurements were made on powder samples in continuous mode, scanning at 0.5°/min over the 2θ range 20–70° and recording the count every 0.02°. The diffractograms were compared with the standard magnetite (Fe_3_O_4_) diffractogram available from the Cambridge Crystallographic Database (CSD).

#### 2.9.2 Magnetic Characterization

Magnetic characterization of the SPIONs samples was conducted using a vibrating sample magnetometer (VSM), which is integrated into a Quantum Design physical property measurement system (PPMS) 9–evercool II. Magnetization (*M*) as a function of the applied magnetic field (H) was measured within a field range of ±1 T at both 5 and 300 K. Additionally, the temperature dependence of magnetization, *M*(*T*), was evaluated using zero-field-cooled (ZFC) and field-cooled (FC) protocols across a temperature range of 5 to 300 K, with an applied magnetic field of 0.1 T. All measurements were mass-normalized to ensure consistency across data.

#### 2.9.3 Zeta Potential (ZP)

The zeta potentials of the SPIONs prepared by synthesis and of the porcine trypsin obtained commercially were determined in the Zetasizer Nano ZS equipment (Malvern Instruments). To determine the PZ of these materials, SPIONs dispersions were prepared at a concentration of 0.07 mg mL^–1^, or porcine trypsin solutions at a concentration of 1 mg mL^–1^, in deionized water with pH values previously adjusted to (ranges from 3 to 13) by simple addition of acid or base. A digital pH meter (model mPA-210) containing a semisolid and liquid shielded pH electrode from Sensoglass, Model SC06 N/S: 00829–15, was used for pH control. These SPIONS dispersions and trypsin solutions were then sonicated for 15 min and filtered through Millex LCR 25 mm, Millipore 0.22 μm filters. For zeta potential measurements, the equipment was configured considering water as a dispersant, viscosity of 0.8872 cP, a refractive index of 1.330, and an electrical conductivity of 78.5, and the temperature was controlled at 25 °C throughout the analyses.

#### 2.9.4 Scanning Electron Microscopy (SEM)

The size and morphology of SPIONs and SPION@PT samples were analyzed by using scanning electron microscopy (SEM). The FEG-SEM microscope was operated at 10 keV with a spot size of 3. Small fragments of samples were placed on a silicon substrate secured with carbon tape and oven-dried at 40 °C for 12 h. Analysis using a 2D energy dispersive X-ray detector (EDS) was performed with 2D mapping at 25 keV and spot 4. The 2D EDS images were generated based on the energy released from the emission of O K_α_ and Fe K_α_.

#### 2.9.5 Infrared Spectroscopy (FTIR)

Infrared spectra for the prepared SPIONs and SPION@PT samples were obtained by using an IR spectrophotometer (Thermo Scientific Nicolet, model IS 50 FT-IR) with a horizontal attenuated total reflectance accessory. Measurements were performed by using a zinc selenide (ZnSe) crystal. The spectra of the samples were obtained, without any previous sample preparation, in the range of 4000 to 500 cm^–1^ with a spectral resolution of 4 cm^–1^ and 32 scans.

## 3 Results and Discussion

### 3.1 Synthesis and Characterization of SPIONs and SPION@PT

In the present work, the traditional method of coprecipitation of Fe^2+^ and Fe^3+^ ions in an aqueous medium by the addition of a base^35^ was used for the preparation of superparamagnetic Fe_3_O_4_ nanoparticles. This method allows for the production of SPIONs under ambient conditions or with minimal temperature increase, resulting in small, high-purity particles with a consistent shape and size. The adjustment of the size and shape of the nanoparticles depends on the establishment of control conditions, such as ion concentration, stoichiometric relationship, solution pH, synthesis time, reducing agent concentration, stirring speed, temperature, among others.^36^ However, in this methodology, there is difficulty in obtaining monodisperse materials because two processes occur simultaneously and need to be controlled to achieve more homogeneous sizes. The first one is nucleation, which consists of the formation of atomic clusters in a homogeneous way, and the phenomenon of growth, which refers to the aggregation of atomic nuclei for the formation of nanoparticles. In general, a reduced particle size dispersion is obtained when nucleation occurs quickly, that is, in a short period of time, followed by a slow growth step without significant nucleation.^37^ Thus, the slow and controlled addition of the reducing agent (base) to the solution containing Fe^2+^ and Fe^3+^ ions is of fundamental importance to obtain nanoparticles with greater homogeneity in size and shape.

The SPION samples were further characterized by XRD and VSM, with the results provided in the Supporting Information (Figures S1 and S2). The X-ray diffraction pattern (Figure S1) confirms the presence of the magnetite crystalline phase (Fe_3_O_4_), with distinct diffraction peaks at 2θ values of 30.2, 35.6, 43.2, 53.65, 57.1, and 62.8°, corresponding to the (220), (311), (400), (422), (511), and (440) planes characteristic of the inverted spinel structure. These values are consistent with the standard magnetite reference from the International Center for Diffraction Data (ICDD).

The magnetic properties of SPIONs, critical to their role in enzyme immobilization systems such as SPION@PT, were investigated through zero-field-cooled (ZFC) and field-cooled (FC) magnetization measurements (Figure S2a). The ZFC and FC curves, measured under a 1000 Oe magnetic field, display a clear irreversibility beginning at a broad peak in the ZFC curve, indicative of the blocking temperature (*T*_B_). This blocking temperature marks the threshold below which the magnetic orientations of individual SPIONs become thermally stable, essentially “locking” their magnetic moments and confirming the superparamagnetic (SPM) behavior characteristic of Fe_3_O_4_ nanoparticles at the nanoscale. Importantly, superparamagnetism implies that at room temperature, the SPIONs can respond rapidly to an external magnetic field, critical for effective magnetic separation, yet return to a nonmagnetized state upon field removal, allowing easy redispersion. This behavior enhances the reusability and efficiency of the SPION@PT system by ensuring it can be magnetically collected and then readily dispersed once the field is removed, thus facilitating repeated catalytic cycles with minimal aggregation.

Further magnetic field-dependent measurements (*M* vs *H*) conducted at 300 and 5 K (Figure S2b) illustrate the distinct temperature dependence of the SPIONs’ magnetic properties. At room temperature (300 K), the magnetization loop approaches saturation at an applied field of around 10,000 Oe (1 T), indicative of typical superparamagnetic behavior with minimal coercivity, allowing rapid alignment and misalignment with applied fields. At 5 K, however, an increased coercive field of 330 Oe and approximately 9% higher saturation magnetization (*M*_s_) are observed. These low-temperature measurements further confirm the stability of the magnetite phase and its ferrimagnetic properties at reduced temperatures, but at 300 K, the SPIONs maintain superparamagnetic behavior, essential for applications requiring fast magnetic response and reversible dispersion. These properties ensure that under typical application conditions matching the optimal activity range of trypsin, the SPION@PT complex can be easily manipulated with a magnetic field without residual magnetization, promoting uniform distribution and high catalytic efficiency.

The morphological and structural characteristics of SPIONs and SPION@PT were evaluated by using SEM. The results presented in this article demonstrate the successful synthesis of superparamagnetic iron oxide nanoparticles (SPIONs) with dimensions smaller than 15 nm. SEM analysis revealed qualitatively spherical nanoparticles consistent with the method applied. These findings are in line with the methodology applied in our research group,^35,38,39^ as well as in similar studies in the literature.^40,41^ Alvarenga et al.^39^ obtained similar results using the same synthesis methodology, using the coprecipitation method in an aqueous medium, which reported an average diameter of 8.3 ± 2.7 nm for SPIONS nanoparticles.

The SEM images demonstrated the homogeneous size of SPION nanoparticles (Figure 2a and Supplementary Figure S3). The histogram from the SEM image (Figure 2a) indicated an average particle diameter of 13.7 ± 4.8 nm. The SEM images also demonstrated the homogeneous size of SPION@PT (Figure 2b and Supplementary Figure S4). The histogram of the SEM image (Figure 2b) indicated a particle diameter of 14.3 ± 4.6 nm. Notably, no significant differences were observed between the SPION and SPION@PT samples (Figure 2a,b), indicating that the addition of trypsin did not significantly alter the particle size. This observation can be explained by considering the relative sizes and volumes of SPIONs and porcine trypsin.

**Figure 2:**
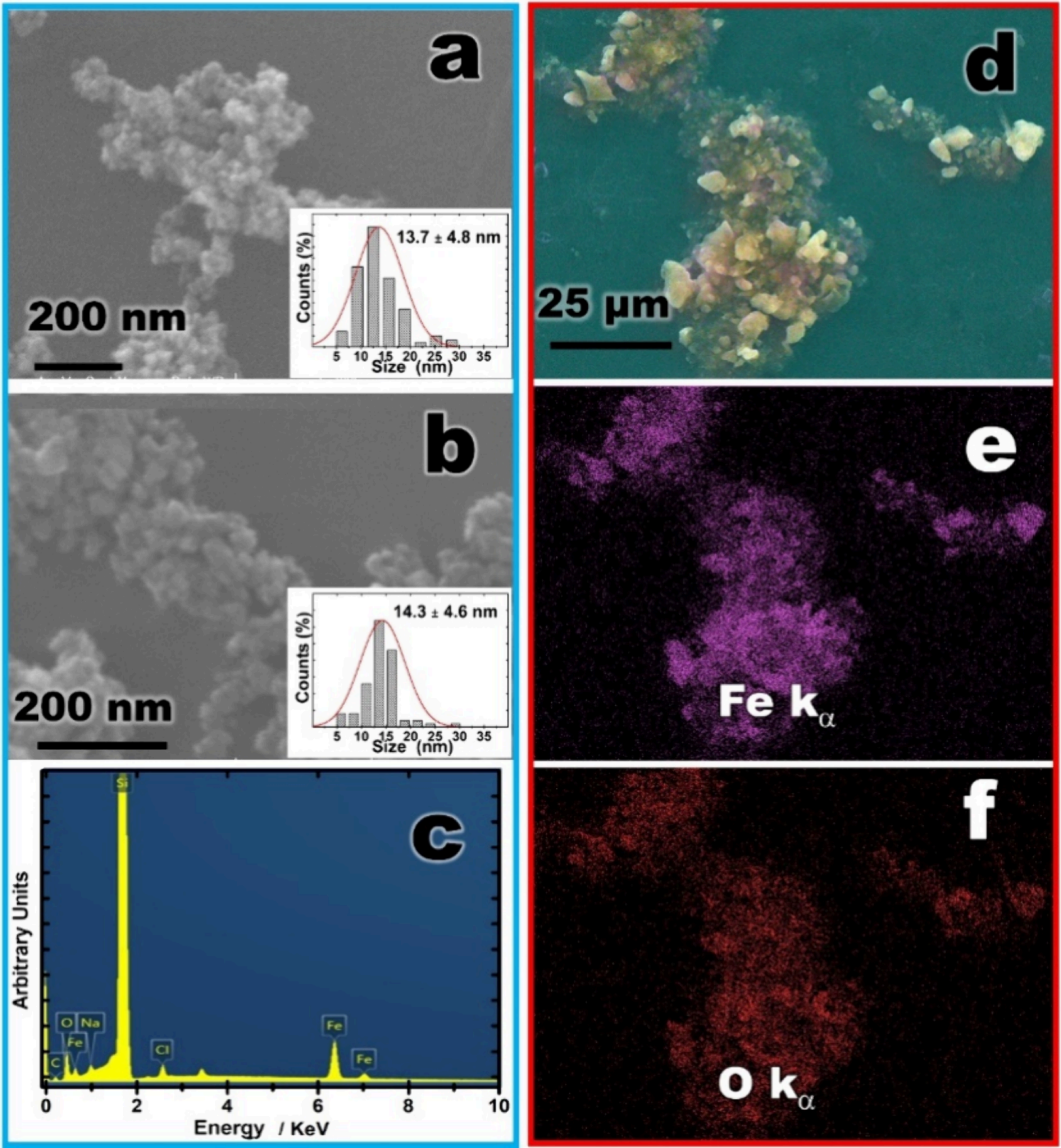
SEM images showing the homogeneous size and distribution of magnetite nanoparticles with a histogram of nanoparticle size distribution. (a) SPIONs; (b) SPION@PT; (c) EDS spectrum showing the presence of Fe and O elements in the SPION@PT; (d–f) EDS elemental mapping of C K_α_ and Fe K_α_ of SPION@PT.

SPIONs have a diameter of approximately 13.7 ± 4.8 nm, giving them a volume of about 1436.76 nm^3^. In contrast, porcine trypsin, when approximated as a sphere, has a diameter of around 3.82 nm and a volume of about 28.8 nm^3^. The significantly smaller volume of trypsin compared to that of SPIONs, with a volume ratio of approximately 1:50, means that the immobilization of the enzyme on the surface of the nanoparticles does not substantially increase the overall particle size. This is evidenced by the observed diameters of 13.7 ± 4.8 nm for SPIONs and 14.3 ± 4.6 nm for the SPION@PT system, indicating a very small increase in volume. The relatively small size of the trypsin molecules allows them to adsorb onto the SPION surface without causing a measurable increase in the nanoparticle diameter, which is why the SEM images show similar sizes for both the SPION and SPION@PT samples. The analysis of SPION@PT showed a similar size to SPIONs, but the particles tended to form clusters after drying the sample, with clusters measuring more than 1 μm. Supplementary Figure S3 shows the distribution of SPION@PT clusters.

The homogeneous distribution of trypsin in the magnetite nanoparticles suggests that the particles were well-dispersed within the material, avoiding excessive concentration in specific areas (Figure 2d–f). The EDS analysis (Figures 2d–f) of the SPION@PT revealed the presence of iron (Fe) and oxygen (O) from SPION nanoparticles (turquoise and red spots, respectively). The 2D images were generated by analyzing the energy released from Fe Kα (Figure 2e) constituents of the SPIONs nanoparticles as well as O Kα emissions (Figure 2f), indicating the uniform distribution of these elements in the demarcated area in the micrograph (Figures S3 and S4). SEM images are unable to distinguish SPION nanoparticles' size due to their proximity, but EDS spectrum analysis makes it possible to confirm the presence of iron oxide in the SPION@PT. Energy Dispersive X-ray Spectroscopy (EDS) (Figure 2c) can identify the presence of specific chemical elements, such as iron and oxygen, and provide information about their distribution in the material. In the region between 0.2 to 1.0 keV (Figure 2c), peaks corresponding to C Kα, O Kα, and Fe Lα emissions are clearly observed at 0.277, 0.525, and 0.705 keV, respectively. In the region between 6 and 7.0 keV, a peak at 6.398 keV corresponding to Fe Kα emissions is visible. The graph indicates the presence of SPIONs, and trypsin demonstrates the occurrence of C, O, and Fe atoms in the SPION@PT sample. Additionally, the EDS spectrum of the composite reveals the presence of Si elements (1.74 keV), originating from the silicon substrate used for sample deposition. The homogeneous distribution of SPIONs suggested that the particles were properly dispersed in the material without excessive concentration in specific areas.

The zeta potential dependence of commercially obtained porcine trypsin and synthesized SPIONs was determined as a function of the pH of the medium (Figure 3).

**Figure 3:**
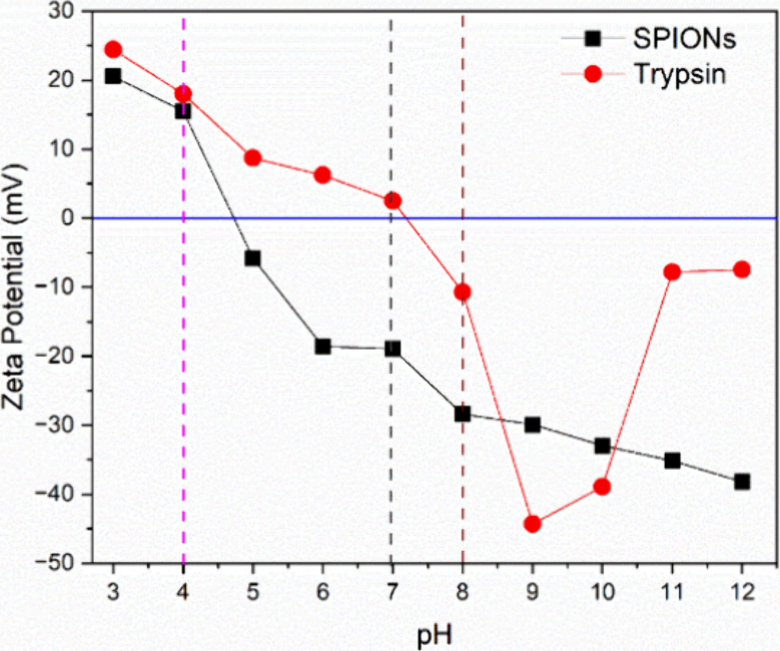
Comparison of experimental values of zeta potential as a function of pH for (- ● -) commercial porcine trypsin and (- ■ -) SPIONs.

The analysis of the results obtained for the measurements of the zeta potential as a function of the pH of the medium for aqueous dispersions of SPIONs and commercial Trypsin solutions revealed, in general, three regions with different promising potentials in providing different interactions between SPION and trypsin. In this sense, for the immobilization studies, pH values of 4.0 were chosen, where SPIONs are found with PZ values of +15.3 mV and Trypsin +17.6 mV; pH 7.0 where the PZ values were +19.2 mV and −2.0 mV, and finally, at pH 8.0 with PZ values of −28.5 and −10.0 mV, for SPIONs and to Trypsin, respectively. Also, the optimal pH range of trypsin, between 7.0 and 8.0, was considered in the immobilization process in order to evaluate the amount of enzyme nonbonded. The more negative zeta potential values of trypsin observed at pH 9 result from the predominance of ionized carboxylate groups (−COO^–^) and possibly the partial deprotonation of amine groups, which confer a more negative net charge to the enzyme. Trypsin, like many proteins, possesses various ionizable functional groups. At pH 9, which is slightly alkaline, most carboxyl groups (−COOH) will be deprotonated, forming −COO^–^, while amine groups (−NH_2_) may remain protonated or begin to deprotonate, forming −NH^–^. The net charge of the protein at pH 9 will be more negative compared to a lower pH, such as pH 7, where the amine groups would be more protonated, and the overall charge would be less negative or even neutral.^42^

Infrared spectroscopy (FTIR) has been widely employed to evaluate enzyme immobilization processes on various supports due to its effectiveness in analyzing structural changes and functional group interactions.^43,44^ In this study, FTIR was used to characterize the functional groups on the surface of synthesized SPIONs, examine the structure of the enzyme (commercial porcine trypsin), and investigate the nanobiocatalyst formed after the immobilization process (SPION@PT), allowing for a comparative assessment of surface modifications following trypsin immobilization. Figure S5 presents the overlaid spectra, enabling the clear observation of the modifications induced by enzyme immobilization.

The infrared spectrum corresponding to the synthesized SPIONs (red line in the upper part of Figure S5) displays bands in the 1600–1200 cm^–1^ region, which are attributed to the stretching and bending vibrations of hydroxyl groups attached to the nanoparticle surface.^20,45^ The band at 571 cm^–1^ corresponds to Fe–O stretching, characteristic of the octahedral and tetrahedral sites in the magnetite (Fe_3_O_4_) structure. Around 2350 cm^–1^, bands are visible across all three spectra, associated with atmospheric CO_2_, likely due to prolonged exposure of the samples to air during spectrum acquisition.

The spectra for porcine trypsin (black curve) and nanobiocatalyst SPION@PT (blue curve) reveal the main vibrational modes characteristic of the enzyme. Peaks at 1745, 1638, and 1199 cm^–1^, corresponding to C=O stretching, N–H bending, and C–O stretching, are observed in free trypsin. However, in the SPION@PT nanobiocatalyst, these C=O and C–O vibrational modes are shifted to 1658 and 1099 cm^–1^, respectively. The N–H bending mode is not visible for the immobilized enzyme, likely due to overlapping hydroxyl group vibrations at around 1535 cm^–1^, which may be attributed to moisture in the sample or the system. The band at 672 cm^–1^ in the enzyme-NP bioconjugate indicates Fe–O stretching, similar to that observed in the pure SPION spectrum. These shifts suggest an electrostatic interaction between trypsin and the nanoparticle surface, as reported in recent studies on similar bioconjugates.^46–49^

### 3.2 Immobilization of Porcine Trypsin in SPIONs

After the immobilization process, the nanoparticles were separated from the supernatant, and the enzymatic activity of SPION@PT at different pH values was measured to evaluate the efficiency of enzyme adsorption on the SPION surface. Table 1 presents the specific activity values of both the immobilized material and its supernatant across the different pH conditions, expressed in both U/mg and katal/mg units. These alternative units facilitate comparison with other studies; however, the discussion will focus on the percentage of recovered activity, which provides a clearer indication of the immobilization efficiency across varying conditions.

**Table 1:** Specific Enzymatic Activity of Free Porcine Trypsin and SPION@PT at Different pH Levels, with Recovered Activity (%) in Both the Immobilized and Supernatant Phases

sample	specific activity (U/mg)	specific activity (katal/mg)	activity recovered (%)
free porcine trypsin	1500.0	2.50 × 10^–5^	100.00
SPION@PT–pH 4.0	477.6	7.96 × 10^–6^	31.84
supernatant–pH 4.0^a^	1252.8	2.09 × 10^–5^	83.52
SPION@PT–pH 7.0	413.4	6.89 × 10^–6^	27.56
supernatant–pH 7.0^a^	1154.1	1.92 × 10^–5^	76.94
SPION@PT–pH 8.0	484.2	8.07 × 10^–6^	32.28
supernatant–pH 8.0^a^	1196.3	1.99 × 10^–5^	79.75

a The supernatant is the final solution obtained after removing the SPION@PT.

The results indicated that trypsin immobilization occurred across all of the adsorption processes studied. However, immobilization at pH 8.0 provided a greater recovery of the activity value compared to the activity value of the enzyme in its free form, although the differences are within the standard deviation range and thus should be interpreted with caution. It was also observed that the supernatants exhibited a high percentage of activity, indicating that a significant amount of enzyme remained in the reaction medium. The higher recovery of trypsin activity at pH 8.0 can be attributed to the optimal ionization state of amino acid residues, which enhances the enzyme’s structural stability and activity. At pH 8.0, the balance between protonated and deprotonated states of the enzyme’s functional groups might contribute to maintaining its active conformation, thus preserving its catalytic efficiency.^50^

The observed behaviors can be explained by understanding that the interactions in the adsorption processes occur across the support surface and are not specifically directed. This consideration makes it difficult to define and control which region of the enzyme will interact with the surface of the support and which residual groups of the amino acids will form the peptide bonds that govern these interactions.

Additionally, the observation that a significant amount of enzyme activity remains in the supernatant can be rationalized by considering the surface properties of the nanoparticles. For a 15 nm iron oxide nanoparticle, approximately 25% of the atoms are on the surface, which serve as anchoring sites. Since each enzyme molecule occupies more than one active site on the support, it reduces the available surface area for the binding of additional enzymes. This results in a substantial proportion of the enzyme remaining unbound in the reaction medium, contributing to the high activity observed in the supernatants.

### 3.3 Effect of Ionic Strength on the Immobilization and Activity of Trypsin

In this study, the effect of ionic strength on the immobilization, desorption, and recovered enzymatic activity of the interaction between SPIONs and porcine trypsin was evaluated. The results obtained are presented in Table 2.

**Table 2:** Effect of Ionic Strength on the Immobilization, Desorption, and Enzymatic Activity of SPION@PT^a^

samples	immobilization	desorption	recovered activity
(ABS concentration)	(%)	(%)	(%)
SPION@PT	70.33 ± 0.97	1.88 ± 0,29	2.54 ± 0.02
(2,5 mM)			
SPION@PT	72.14 ± 0.31	3.13 ± 0,15	19.55 ± 0.10
(25.0 mM)			
SPION@PT	88.16 ± 0.57	3.17 ± 0,32	26.73 ± 0.19
(50.0 mM)			
SPION@PT	99.05 ± 0.04	1.08 ± 0,19	16.50 ± 0.07
(100.0 mM)			

a The study was conducted by adding 0.01 g of porcine trypsin to 20 mL of acetate buffer solution (ABS) at pH 4.0 under different ionic strength conditions (2.5, 25.0, 50.0, and 100.0 mM).

Ionic strength can affect enzyme activity by altering its stability and solubility, as well as that of the substrate. The modulation of enzymatic activity as a function of changes in the ionic strength of the medium generally occurs due to the neutralization effect of counter-ions and the influence on the p*K*_a_ values of ionizable residues necessary for substrate binding, thereby affecting their electrostatic interactions. This effect can alter protein conformation, affecting the exposure of active residues and, consequently, catalytic activity.

The results in Table 2 demonstrate that the immobilization process can be enhanced with an increase in ionic strength from 2.5 to 100 mM, achieving an efficiency of 99% in the immobilization of trypsin on SPIONs. A low desorption rate was observed in all ionic strengths analyzed, remaining below 3.2%, indicating that the interaction between the enzyme and the support occurs through strong forces, as described by Cavalcanti and co-workers.^51^ The recovered enzymatic activity shows that increasing the ionic strength to 50 mM significantly enhances the enzymatic activity. However, the results indicate that the mobility of the counter-ions in solution does not modify the medium significantly enough for the change in ionic strength to favor a different organization of trypsin on the surface of SPIONs, thereby improving the recovered enzymatic activity after the immobilization process, as described in Section 3.2. Thus, this study suggests that many of the solvent molecules bound to the protein occupy similar areas in the presence of low, medium, or high ionic strength. The observed low desorption even at higher salt concentrations can be explained by changes in the protonation states of surface groups, which modulate electrostatic interactions. These stronger interactions, as described in da Silva Cavalcanti et al.,^27^ promote stable enzyme binding and reduce leaching.

### 3.4 SPION-PT Interaction

To determine the amount of enzyme adsorbed on the surface of the nanoparticles, a fluorescence spectroscopy method was used, which is based on the intrinsic fluorescence of biological macromolecules. The results regarding the protein content in the supernatant of the immobilized material are presented in Table 3. Fluorescence analysis showed that a portion of the enzyme remained in the supernatants, indicating that 66.2% trypsin was adsorbed onto the SPIONs at pH 4.0, 67.1% at pH 7.0, and 75.4% at pH 8.0. In all three cases, therefore, the immobilization process was satisfactory.

**Table 3:** Analysis of Protein Content in the Immobilization Process Supernatant^a^

sample	concentration (μg mL^–1^)
trypsin	4.60 (100%)
S. SPION@PT, pH 4.0	1.55 (33.8%)
S. SPION@PT, pH 7.0	1.516 (32.9%)
S. SPION@PT, pH 8.0	1.13 (24,6%)

a S. SPION@PT refers to the supernatant of the immobilized material.

As the interaction between enzyme and support can occur through weaker interactions that favor the desorption of the enzyme immobilized on the surface of the nanoparticles, the process of mechanical desorption of the synthesized material when exposed to an ultrasonic washer for 5 cycles of 5 min each was analyzed and monitored by fluorescence spectroscopy.

#### 3.4.1 Isothermal Titration Calorimetry (ITC) Results

ITC is an invaluable technique for studying the interaction between SPIONs and Trypsin because it directly measures the heat changes associated with binding, providing comprehensive thermodynamic parameters.^52^ This technique captures the differential power required to maintain the temperature of the sample cell, containing SPIONs, equal to the reference cell as trypsin is injected, which allows, after data treatment, the construction of binding isotherms (apparent enthalpy change (Δ*H*_ap_) vs molar ratio), as shown in Figure 4.

**Figure 4:**
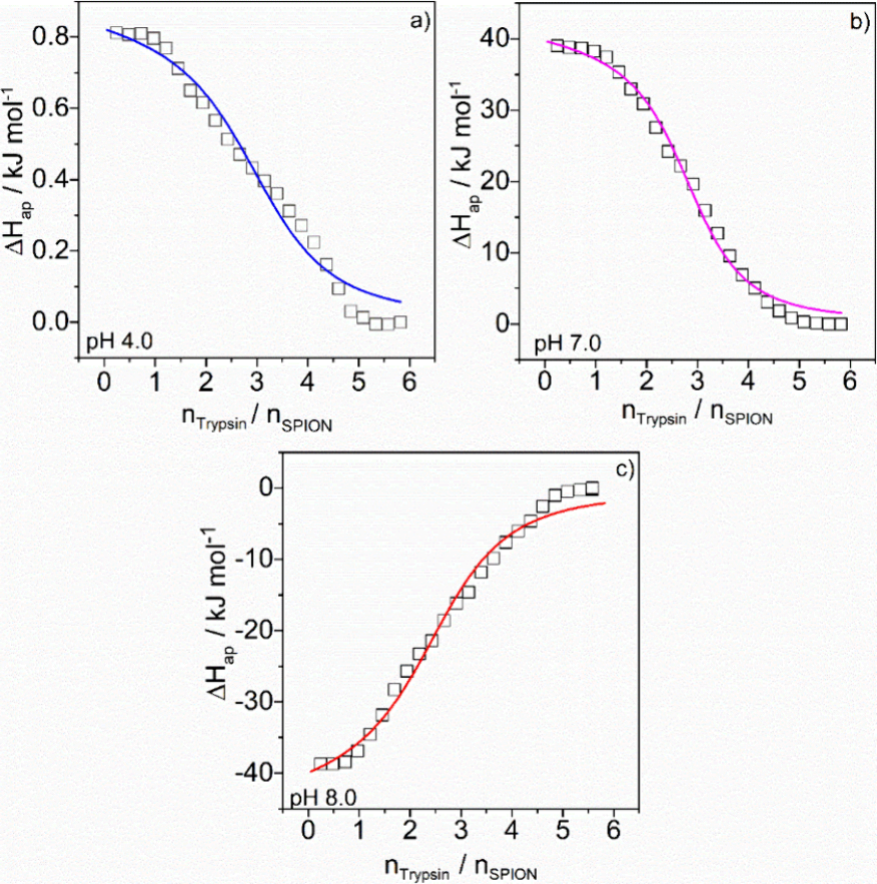
Binding isotherms for the SPION-PT interaction at 298.15 K under different pH conditions: (a) 4.0, (b) 7.0, and (c) pH 8.0. Square symbols represent experimental data, and the solid line indicates the fitting obtained using the SSIS model.

The binding isotherms for the SPION-PT interaction at pH values of 4.0, 7.0, and 8.0 fit well to the SSIS model, allowing the determination of *K*_b_, *n*, and Δ*H*° (eqs 1 and 2), and consequently the calculation of Δ*G*° (eq 3) and *T*Δ*S*° (eq 4), as shown in Figure 5.

**Figure 5:**
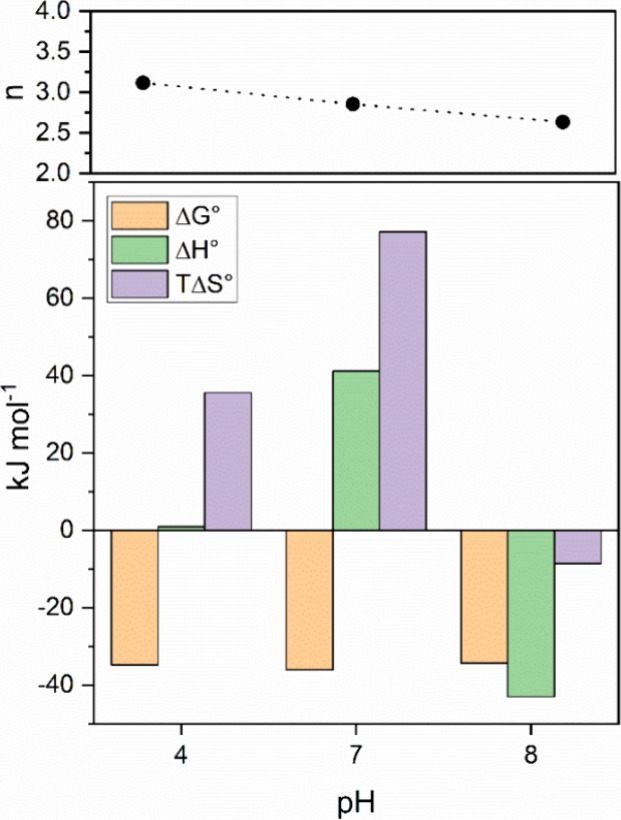
Thermodynamic parameters for the binding of trypsin to SPION at 298.15 K under different pH conditions.

At all studied pH values, the obtained stoichiometry indicated the binding of three mol of trypsin per mole of SPION. The Δ*G*° values obtained were negative, showing that the formation of the SPION@PT complex is favored in the thermodynamic equilibrium. Moreover, although the stability of the SPION@PT complex formed was practically independent of the pH, the enthalpic and entropic contributions varied considerably.

At both pH 4.0 and 7.0, the binding of trypsin to SPIONs was predominantly entropy-driven, as indicated by Δ*H*° values of 0.9 and 41.1 kJ mol^–1^, and *T*Δ*S*° values of 35.6 and 77.2 kJ mol^–1^, respectively. These observations indicate that the binding process is substantially driven by the release of water molecules from the solvation layers around the protein and SPIONs, along with conformational changes in the protein structure. These factors outweigh the energy contributions from the formation of noncovalent interactions, primarily van der Waals forces.

Zeta potential measurements revealed that both trypsin and SPIONs are positively charged at pH 4.0, leading to electrostatic repulsion, which potentially obstructs effective enzyme orientation and binding. Conversely, at pH 7.0, Trypsin exhibits a positive zeta potential, while SPIONs are negatively charged, resulting in attractive electrostatic interactions that promote closer proximity between the protein and the nanoparticle. This electrostatic facilitation likely induces greater conformational changes in the Trypsin molecules upon binding to SPIONs, as evidenced by the more pronounced endothermic Δ*H*° and higher *T*Δ*S*° values. These findings correlate with the observed lower activity recovery at pH 7.0, suggesting significant conformational adjustments of the protein during the binding process.

In contrast, at pH 8.0, the interaction is exothermic with a Δ*H*° of −43.0 kJ mol^–1^ and exhibits a negative *T*Δ*S*° of −8.6 kJ mol^–1^. The exothermic nature indicates that strong noncovalent interactions dominate at this pH. The negative entropy change implies a decrease in the degrees of freedom upon binding, likely due to the specific structural constraints and tighter binding interactions that occur under basic conditions. Despite the negative zeta potentials of both PT and SPIONs at this pH, which typically lead to electrostatic repulsion, the strong hydrophobic interactions dominate, resulting in effective binding. Indeed, the electrostatic surface of Trypsin, presented in Figure 5, demonstrates that at pH 8, the protein exhibits more hydrophobic regions compared to the other pH values. This is corroborated by the highest activity recovery observed at pH 8.0, indicating that the enzyme retains its structural integrity and functionality more effectively under basic conditions.

#### 3.4.2 Molecular Modeling Results

In the theoretical part of this work, we investigated the interaction between porcine trypsin and a magnetite (Fe_3_O_4_) surface by using molecular modeling techniques. Although the Fe_3_O_4_ support was not explicitly modeled as a 3D structure, the enzyme was computationally approached toward a virtual plane that represents the support surface, allowing us to estimate the interaction energies and orientations. The potential energy profiles for adsorption were obtained via semiempirical PM6 calculations, simulating the enzyme approaching the surface from either the side opposite to or including the active site, at different pH conditions.

Figure 6 shows the graphs of adsorption energy profiles at different pH levels and enzyme orientations. According to Figure 6 (graphs A–C), we have the energy profiles for adsorption on Fe_3_O_4_ through approaching the enzyme from the opposite side to the active site. The surface properties of Fe_3_O_4_ (magnetite) vary significantly with pH due to changes in protonation and deprotonation of surface functional groups, which influence adsorption behavior, surface charge, and interactions with biomolecules.

**Figure 6:**
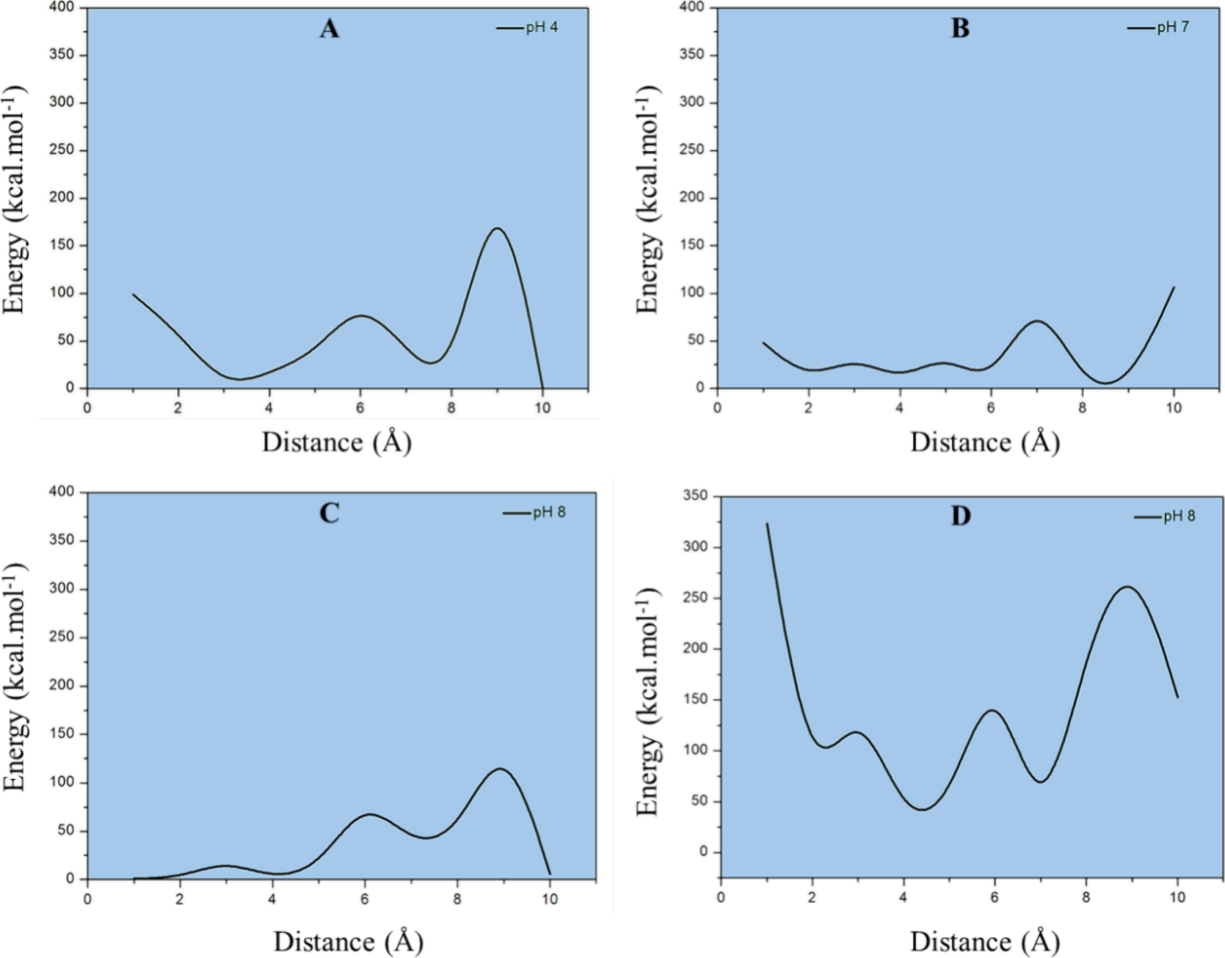
Potential energy profiles for trypsin adsorption onto Fe_3_O_4_. (A–C) Energy profiles for the enzyme approaching the surface from the side opposite to the active site at pH 4, 7, and 8, respectively. (D) Profile for the enzyme approaching the surface from the same side as the active site at pH 8.

The profile shown in Graph 6A indicates the adsorption energy simulated at pH 4. From this result, the energy profile has large energy variations, starting from a distance of 10 Å. Minimum energy values are found at a distance of 3 Å, with the energy rising quickly at distances less than 3 Å. This outcome suggests that the adsorption process and immobilization of porcine Trypsin on Fe_3_O_4_ are unstable, that is, less favorable in comparison with pHs 7 and 8. At acidic pH (pH 4), the Fe_3_O_4_ surface becomes positively charged due to protonation, which may result in strong electrostatic repulsion with the enzyme and weaker adsorption. At this pH, the enzyme carries a net positive charge due to protonation of amino acid residues such as lysine and arginine. However, other interactions, such as hydrogen bonding and van der Waals forces, may still contribute to the adsorption.

On the other hand, for pH 7 (graph 6B), the energy oscillates from large distances, but it tends to remain more constant at low energy values (under 50 kcal mol^–1^) at distances less than 6 Å. In this case, the energy starts to rise again at distances less than 2 Å. Our results suggest that the adsorption at pH 7 is much more favorable than that at pH 4, and therefore, the enzyme seems to be more strongly immobilized on the material surface. As previously discussed, at neutral pH (pH 7), trypsin exhibits a positive zeta potential, while SPIONs possess a negative charge. This charge difference creates attractive electrostatic interactions, promoting closer contact between the protein and the material surface. Intermolecular interactions, such as hydrogen bonding and van der Waals forces, also help stabilize the system, resulting in more stable adsorption energies compared to the system under acidic conditions.

In addition, the same simulation was performed at pH 8 (graph 6C), which gave us the best results for these computational simulations. For this pH, in the same way, it was observed that large variations in energy occurred along large distances. However, the energy decreases and remains stable at very low energy values (distances less than 4 Å), suggesting that the adsorption process and enzyme immobilization are much more favorable and stable, in comparison with pHs 4 and 7. At alkaline pH (pH 8), the surface becomes negatively charged due to deprotonation (Fe–O^–^). In this case, hydrophobic interactions primarily stabilize the system. Therefore, porcine Trypsin, which possesses a negative zeta potential at pH 8, is likely to be more strongly adsorbed onto the material surface due to the presence of hydrophobic interactions.

The orientation of trypsin on the Fe_3_O_4_ surface significantly influences its enzymatic activity. When the active site faces Fe_3_O_4_ (graph 6D), steric hindrance occurs, imposing higher steric constraints that restrict substrate access and reduce the enzymatic efficiency. In contrast, an optimal orientation, where the active site is positioned away from the Fe_3_O_4_ surface, preserves the catalytic efficiency by keeping the active site exposed. Furthermore, pH also plays a crucial role in the Fe_3_O_4_-trypsin interaction, with pH 8 being the most favorable condition, ensuring strong adsorption while maintaining enzyme activity. Conversely, at pH 4, weak adsorption occurs due to electrostatic repulsion.

To further explore the pH-dependent interaction potential, we analyzed the electrostatic surfaces of trypsin at pH 4, 7, and 8 (Figure 7). The electrostatic potential maps show that at pH 4, positively charged regions (blue) dominate near the active site. At pH 7, more neutral and negatively charged areas began to emerge. At pH 8, the predominant regions around the active site are negatively charged (red), which may facilitate stronger electrostatic interactions with positively charged groups on the SPION surface (e.g., Fe^3+^-OH_2_^+^ sites), favoring immobilization.

**Figure 7:**
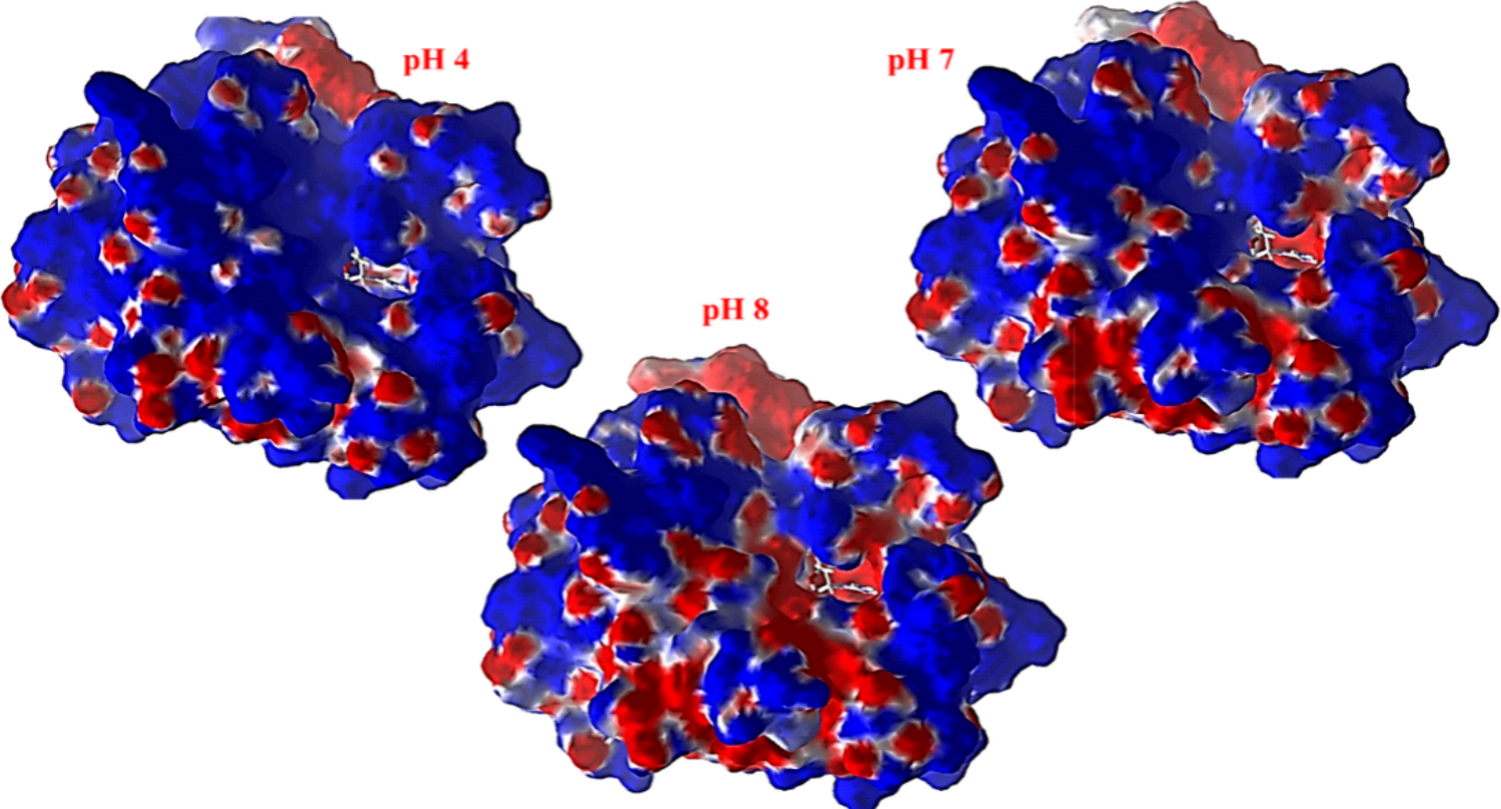
Electrostatic surface potential maps of porcine trypsin at different pH values. Blue regions represent areas of positive charge (less likely to bind to positively charged surfaces), and red regions represent areas of negative charge, which are more favorable for adsorption onto the positively charged support surfaces.

These theoretical results align well with the experimental findings, which revealed enzyme adsorption efficiencies of 66.2, 67.1, and 75.4% at pH 4.0, 7.0, and 8.0, respectively. Therefore, our modeling supports the conclusion that enzyme-surface interactions are significantly enhanced at higher pH due to more favorable electrostatic and steric conditions.

### 3.5 Desorption Studies and SPION-PT Interaction

Desorption studies revealed that after 5 washing cycles, the desorption rate could not be determined, as the values were below the detection limits of the fluorescence calibration curve (0.4 mg mL^–1^). The low desorption rate observed experimentally can be attributed to the occurrence of stronger SPION-PT interactions, perhaps of an ionic character. Ionic adsorptions occur similarly to physical adsorption but involve stronger interactions than Van der Waals forces or hydrogen bonds, although they are weaker than the covalent bonds characteristic of chemical adsorption.^53^

### 3.6 Evaluation of the SPION@PT Reuse Cycle

The ability to reuse the enzyme is a major advantage of immobilization, making it crucial to evaluate its reuse capacity. The reuse of immobilized Trypsin was tested over several cycles in catalytic processes using BapNA as a substrate. In each step, the stability of the nanobiocatalyst was verified from its recovered activity. According to the results presented in Figure 8, the catalysis process occurred with an efficiency of approximately 88% until the second cycle, and the immobilized enzyme retained an efficiency of around 40% until the fourth cycle. This result shows that the nanobiocatalyst maintained its activity after cycles of use, even though it suffered a catalytic loss during the process. This decrease in activity after a few cycles of reuse can be due to the movement of the enzyme itself in its interaction with the substrate, and with each adaptation carried out, a disruption of its protein chain may occur. Despite the decrease in activity, there was a stability of the biocatalysts after the successive cycles, which may be due to an intense interaction of the Trypsin molecules with the support surface, which could reduce possible desorption, as confirmed in the desorption studies. performed.

**Figure 8:**
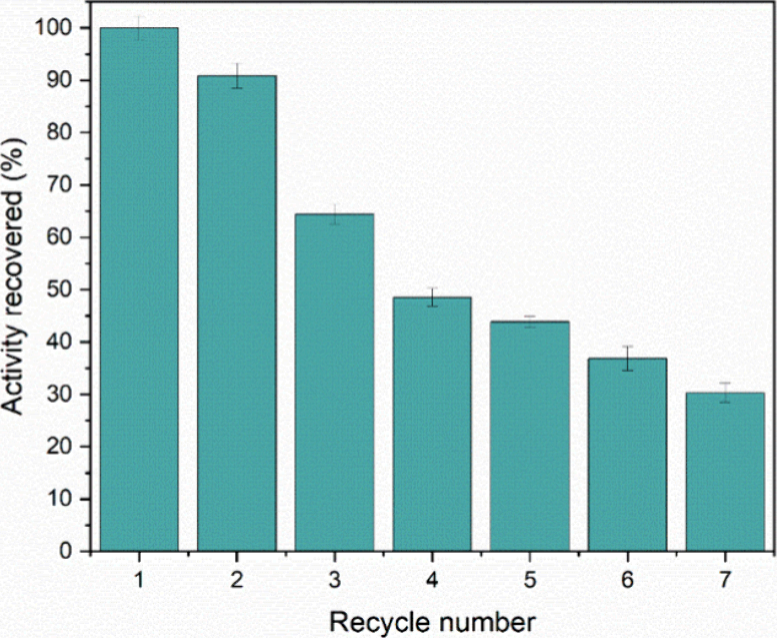
SPION@PT nanobiocatalyst reuse cycle assay.

These results confirm that the enzyme immobilized on a solid support is active and stable for use for several cycles, being economically advantageous in relation to the soluble enzyme, which does not present the possibility of reuse. From this evaluation, it is also possible to affirm the promising use of biocatalysts prepared with magnetic supports due to their ease of separation from the reaction mixture and reuse after successive cycles.

These results can be compared with those of other similar studies, such as Souza Júnior et al.^54^ evaluated the effect of the method of immobilization of Trypsin in biochar on the hydrolysis of casein from different sources, obtaining, in the immobilization by adsorption, the possibility of reusing the use of the biocatalyst for 7 cycles. The activity of the biocatalyst in the hydrolysis of Bovine Casein dropped from 90% in the second cycle to 30% in the seventh cycle of use. Recently, Aggarwal and Ikram^55^ immobilized trypsin on the surface of glutaraldehyde (GA)-activated ZnO/chitosan nanocomposite and stated that the system sustained almost 50% of the catalytic activity until the ninth repeated cycle of use. Peiman et al.^56^ evaluated the use of trypsin bound to the Fe_3_O_4_@SiO_2_@D-NHCS support and stated that the catalyst obtained stability for 5 cycles. Aslani et al.^57^ used Trypsin in Fe_3_O_4_@SiO_2_–NH_2_, which retained its initial activity around 85% for 6 cycles. However, in all of these studies, at least one intermediate treatment step was carried out on the surfaces of the nanoparticles, which increases the preparation time of the nanobiocatalyst and the cost of the process, in addition to its complexity.

Although a gradual loss of enzymatic activity was observed over successive cycles, which is a common challenge in immobilized enzyme systems, the results still demonstrate a reasonable operational stability. When combined with the favorable immobilization conditions supported by thermodynamic and molecular modeling data, these results contribute to a deeper understanding of enzyme-support interactions and provide guidance for further optimization.

## 4 Conclusions

This study provides significant advancements in the field of enzyme immobilization, particularly through the integration of a comprehensive energetic evaluation with conventional adsorption studies. The synthesis of superparamagnetic iron oxide nanoparticles (SPIONs) and their use as supports for the immobilization of porcine trypsin (PT) was systematically explored. Detailed characterizations confirmed the successful synthesis and functionalization of SPIONs, while isothermal titration calorimetry (ITC) and PM6 calculations revealed the thermodynamic favorability of enzyme adsorption at pH 8.0, where the interaction was predominantly exothermic (Δ*H*° of −43.0 kJ mol^–1^). The pH control conditions were previously selected based on extensive experimental (zeta potential, ITC, fluorescence) and computational studies of the materials involved. This pH condition also yielded the highest enzyme activity recovery with over 75% retention of catalytic efficiency, maintaining 40% activity over four reuse cycles.

The energetic evaluation highlighted in this study is a crucial aspect that is often overlooked in enzyme immobilization processes. It not only enhances the understanding of enzyme-support interactions but also provides a strategic framework for optimizing immobilization conditions, potentially reducing costs and improving the technical feasibility of biocatalytic systems. By demonstrating that energy profiles can predict the stability and performance of immobilized enzymes, this work establishes a new standard for the design and application of nanobiocatalysts.

## Supplementary Material



## Notes

Complementary experimental data and characterization figures that support the findings of this study, including X-ray diffraction (XRD) pattern of synthesized SPIONs compared to a standard magnetite reference; magnetic characterization of SPIONs, including (a) temperature-dependent magnetization curves and (b) magnetization versus magnetic field at 5 and 300 K; SEM images and particle size distribution histograms of SPIONs, along with EDS elemental mapping of Fe and O; SEM images and particle size distribution histograms of SPION@Trypsin (SPION@PT), including EDS elemental mapping of Fe and O; and FTIR spectra of synthesized SPIONs, commercial porcine trypsin, and the SPION@PT nanobiocatalyst, illustrating structural and surface modifications upon enzyme immobilization (PDF)
