# Supplementary material for: Immobilization of Porcine Trypsin in Superparamagnetic Nanoparticles: Enzyme Activity and Stability
Source: ACS Omega. 2025 Jun 2;10(22):22970–83. doi: 10.1021/acsomega.5c00797 (PMC12163697; doi:10.1021/acsomega.5c00797)
Supplement: Supplementary file 1 [file ao5c00797_si_001.pdf]

Supporting Information

Immobilization of porcine trypsin in superparamagnetic nanoparticles: enzyme activity and stability

Isabella F. S. Aversa[1], Marcello H. S. Cavalcanti[1]; Thalles M. Pereira[1], Alexandre A. de Castro[2]; Olga L. Tavano[1], Yara L. Coelho[1], Luis H. M. da Silva[3], Luiz F. Gorup[,4];Teodorico C. Ramalho[2], Luciano S. Virtuoso[1]*

[1] Colloid Chemistry Group, Chemistry Institute, Federal University of Alfenas (UNIFAL-MG), 700 Gabriel Monteiro da Silva street, 37130-000 Alfenas-MG, Brazil

[2] Department of Chemistry, Federal University of Lavras, 37200-000, Lavras-MG, Brazil.

[3] Advanced Thermokinetics of Molecular Systems (ATOMS) Group, Department of Chemistry, Federal University of Viçosa, PH Rolfs, Viçosa, MG 36570900, Brazil.

[4] Department of Chemistry, Federal University of Juiz de Fora (UFJF), Juiz de Fora- MG 36036-900, Brazil.

* Corresponding author. luciano.virtuoso@unifal-mg.edu.br

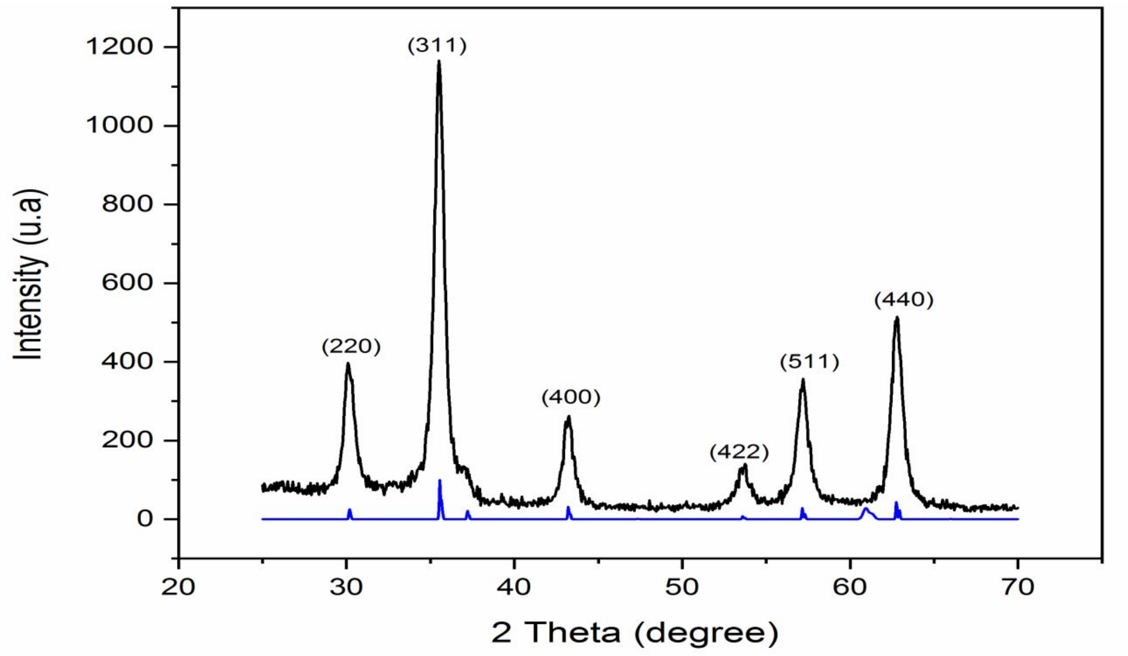

Figure S1. XRD of synthesized SPIONs (-■-) and Magnetite Standard (-■-)

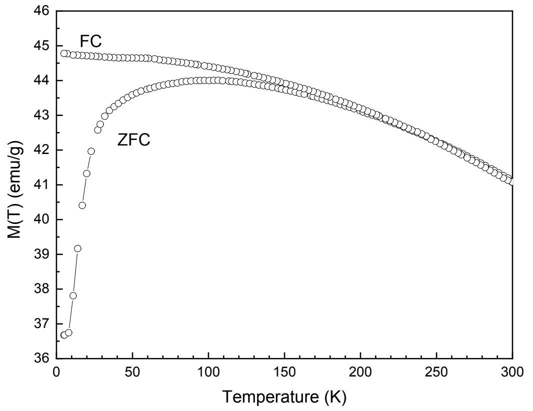

(a)

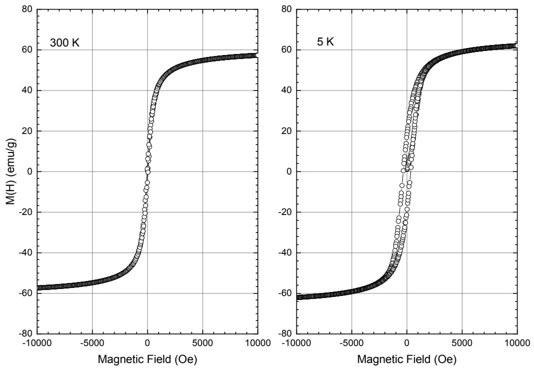

(b)

Figure S2 – (a) Temperature dependence of magnetization and (b) Magnetization versus magnetic field taken at 5K and 300 K of SPIONS.

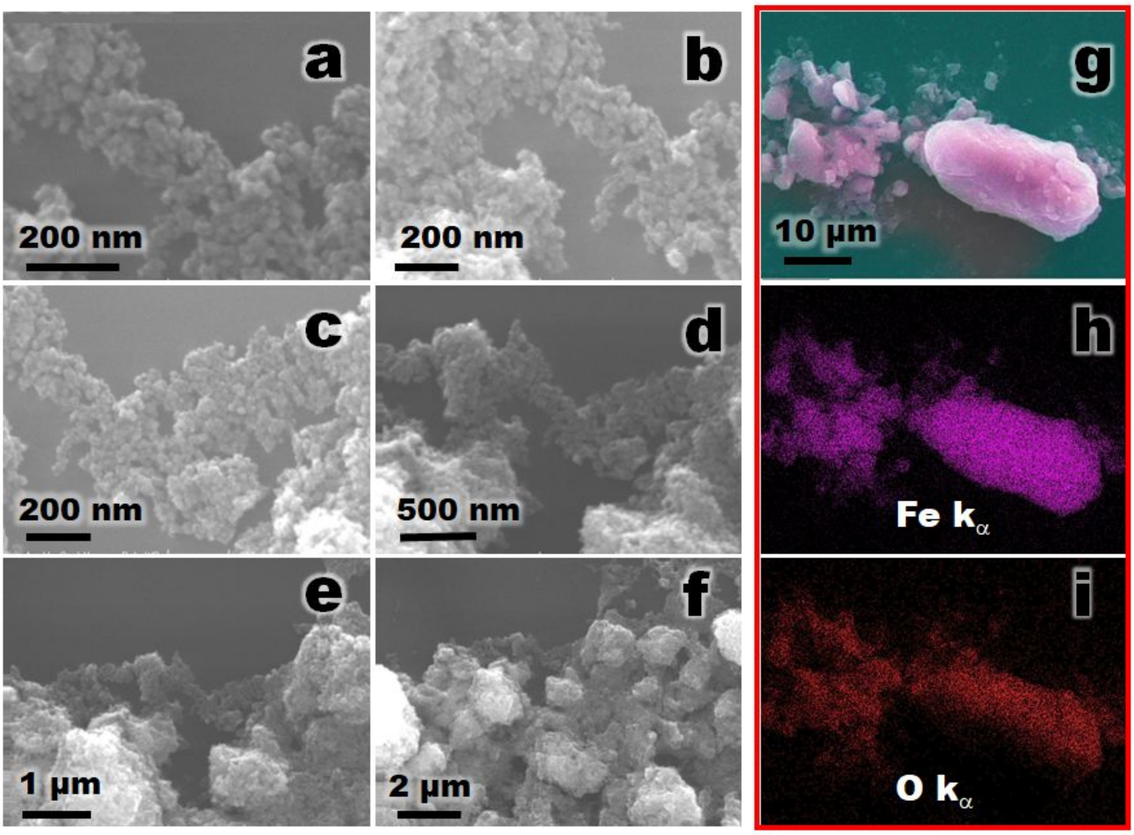

Figure S3- SEM images showing the homogeneous size and distribution of magnetite nanoparticles with the histogram of nanoparticle size distribution. A-f) *SPIONs* at different magnifications, showing the homogeneous size of nanoparticles; g-i) EDS elemental mapping of O Kα and Fe Kα of *SPIONs*

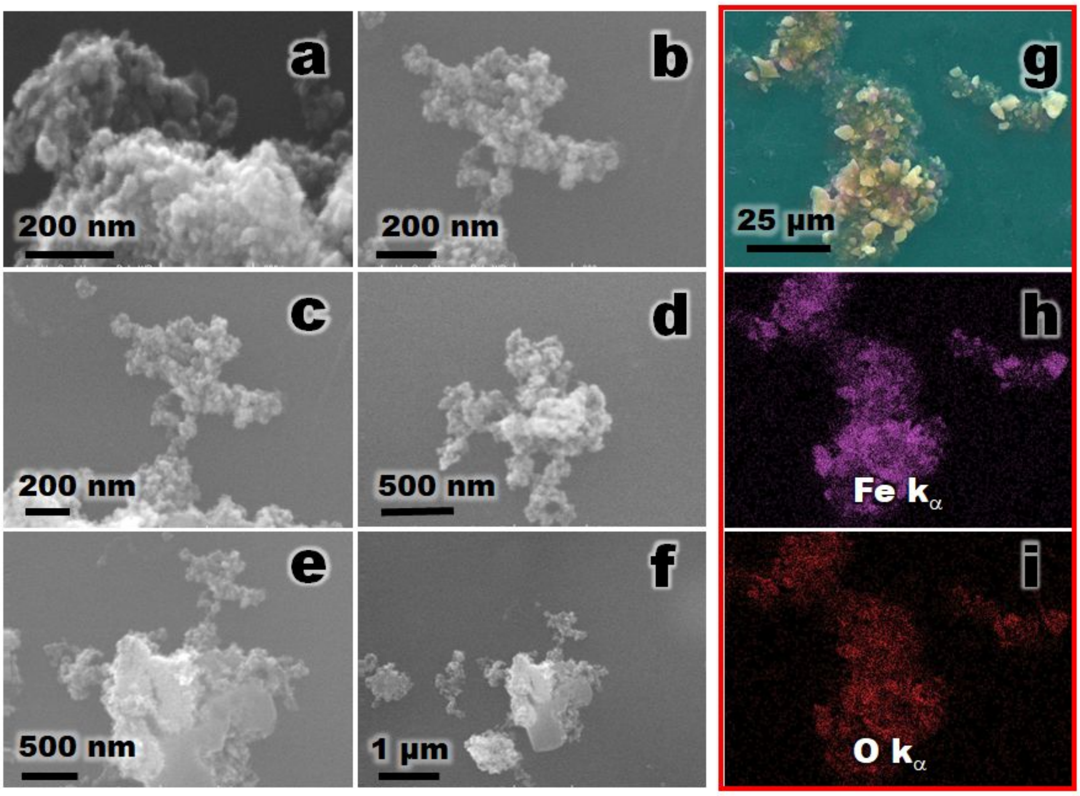

Figure S4 - SEM images showing the homogeneous size and distribution of magnetite nanoparticles with the histogram of nanoparticle size distribution.; a-f) *SPIONs@Trypsin* at different magnifications, showing the homogeneous size of nanoparticles; g-i) EDS elemental mapping of O Kα and Fe Kα of *SPIONs@Trypsin*

The quality of the composites was analyzed using Energy Dispersive Spectroscopy (EDS). The EDS analysis revealed the presence of iron (Fe) and oxygen (O) from magnetite nanoparticles indicated by red and blue spots, respectively. The 2D images were constructed by analyzing the energy emitted by the Fe Kα and O Kα constituents of the magnetite material. These results demonstrate the uniform distribution of these elements in the designated area of the micrograph.

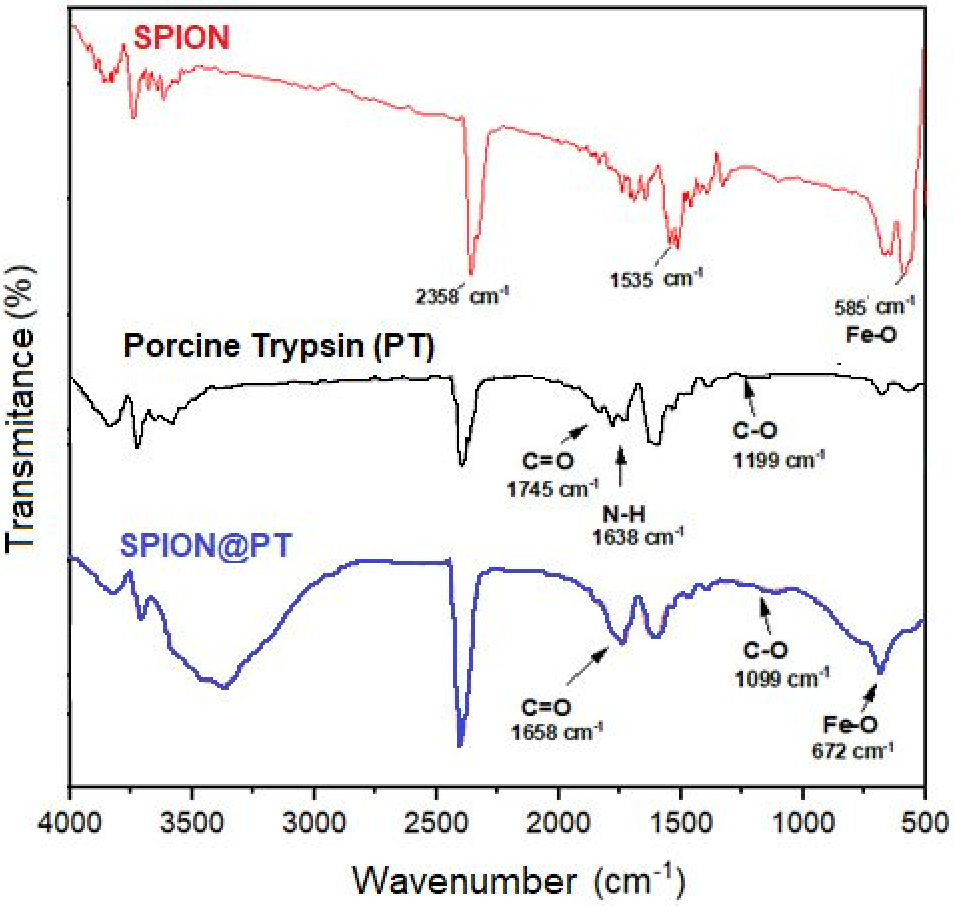

Figure S5 - Infrared spectra of (—) synthesized SPIONs, (—) comercial Porcine Tryspin (PT), and (—) SPION@PT.
